# Supplementary material for: Screening of Tumor Antigens and Construction of Immune Subtypes for mRNA Vaccine Development in Head and Neck Squamous Cell Carcinoma
Source: Biomolecules. 2022 Dec 31;13(1):90. doi: 10.3390/biom13010090 (PMC9855883; doi:10.3390/biom13010090)
Supplement: Supplementary file 1 [file biomolecules-13-00090-s001.zip › biomolecules-2069744-supplementary.pdf]

Screening of tumor antigens and construction of immune subtypes for mRNA vaccine development in head and neck squamous cell carcinoma

Hong-Xia Li[1], Tian-Run Liu[1], Zhao-Xu Tu[2], Chu-Bo Xie[1], Wei-Ping Wen[3,4,1*], Wei Sun[3,4*]

[1] Department of Otorhinolaryngology Head and Neck Surgery, The Sixth Affiliated Hospital, Sun Yat-sen University, Guangzhou, Guangdong 510655, China.

[2] Guangdong Institute of Gastroenterology, The Sixth Affiliated Hospital, Sun Yat-sen University, Guangzhou, Guangdong 510655, China

[3] Department of Otolaryngology, The First Affiliated Hospital, Sun Yat-sen University, Guangzhou, Guangdong, China

[4] Otorhinolaryngology Institute, Sun Yat-sen University, Guangzhou, Guangdong, China

* Correspondence: Wei Sun, *E-mail*: sunwei26@mail.sysu.edu.cn, And Wei-Ping Wen, *E-mail*: wenwp@mail.sysu.edu.cn

Supplemetary Figures and Figure Legends

Figure S1

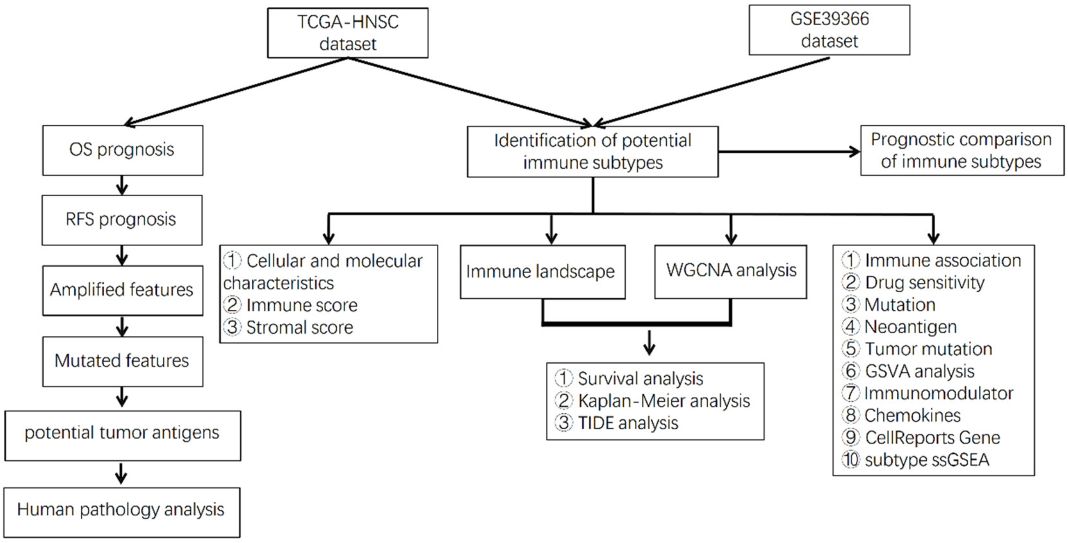

Figure S1. Flowchart diagram of the analysis process of this study.

Figure S2

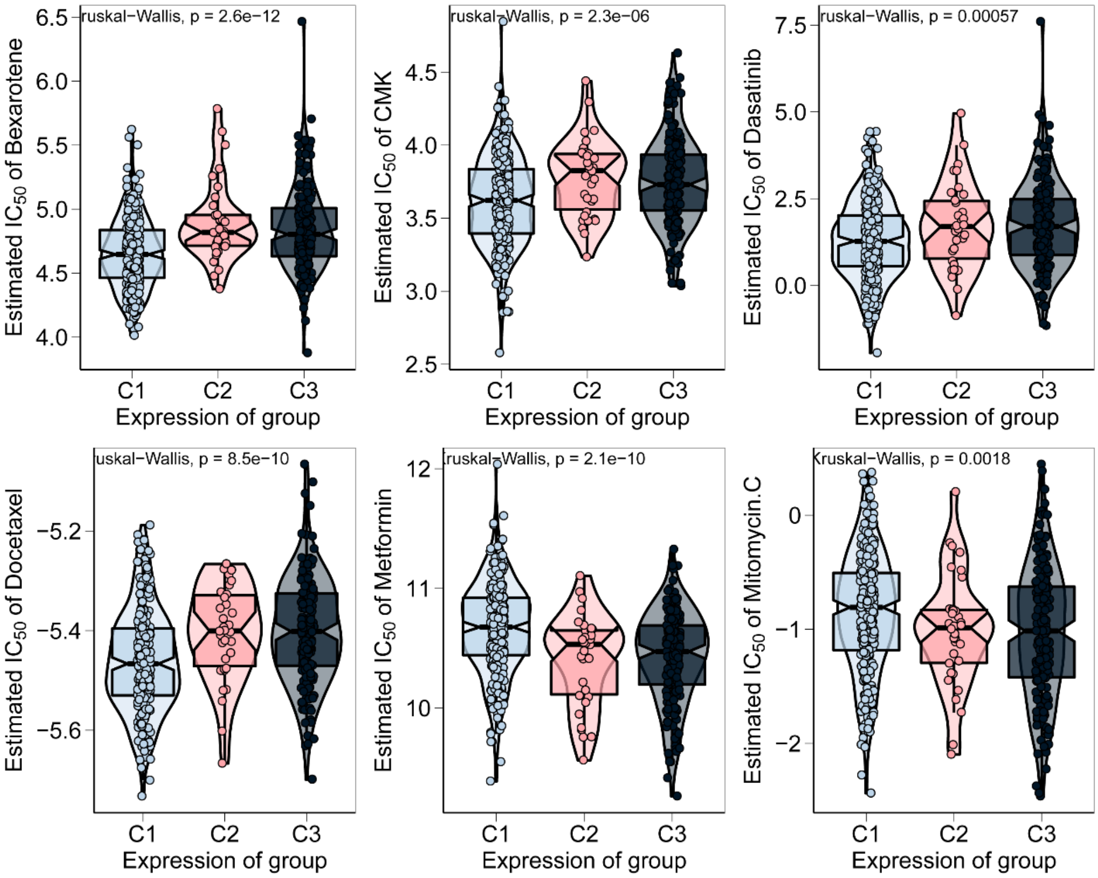

Figure S2. Drug sensitivity analysis among immune subtypes.

Figure S3

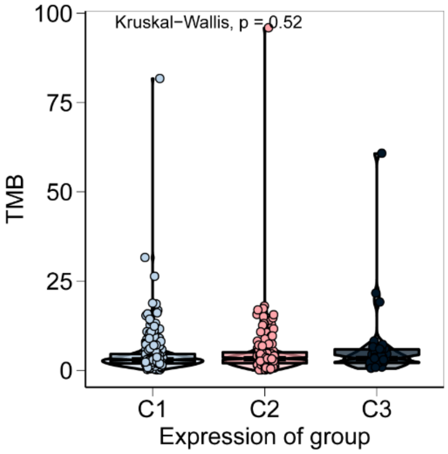

Figure S3. The TMB between expression groups of subtypes

Figure S4

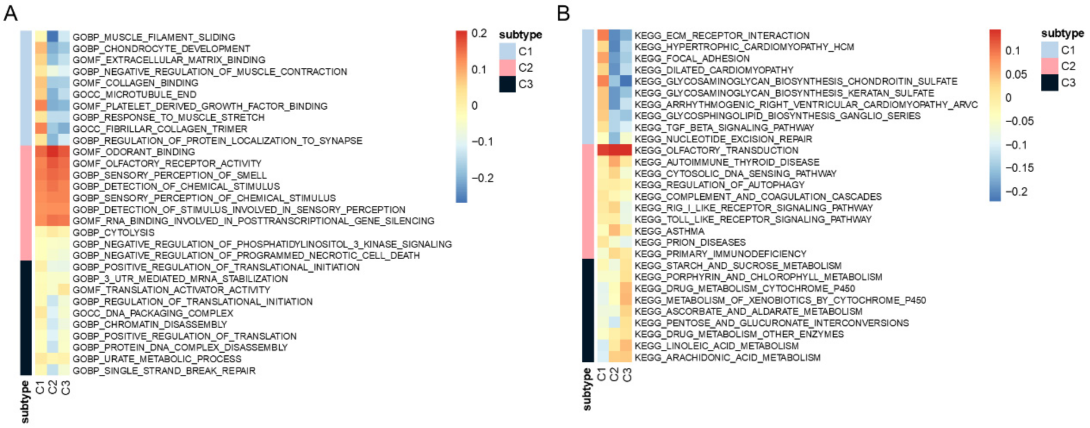

Figure S4. Enrichment analysis of immune subtype related pathways. GO analysis (A) and KEGG analysis (B) of subtype-related ssGSEA.

Figure S5

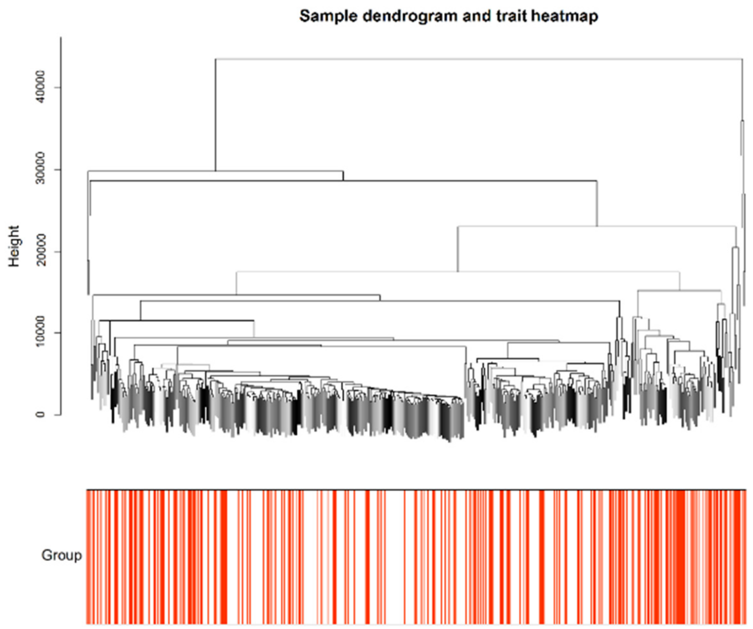

Figure S5. The clustering diagram of HNSCC sample.
